# Is the “Bacillus Lepræ” a Reality or a Fiction?

**Published:** 1882-04

**Authors:** H. D. Schmidt

**Affiliations:** Pathologist of the Charity Hospital, of New Orleans, La.


CHICAGO MEDICAL

JOURNAL & EXAMINER.

Vol. XLIV.—APRIL, 1882.—No. 4.

### Original Communications.

### Article I.

**Is the "Bacillus Lepræ" a Reality or a Fiction? A Critical Review by Dr. H. D. Schmidt, Pathologist of the Charity Hospital, of New Orleans, La. Read before the State Microscopical Society of Illinois.**

The question whether the pathological changes, observed in the various tissues and organs of fatal cases of leprosy, were originally caused by parasitic organisms, is not entirely new, but dates back to 1870, when Hansen published his first observations on the subject. These observations, however, failed to be generally corroborated by other pathologists, who, also, had ample opportunities of examining the tissues of lepers, until 1879. Neisser directed anew the attention to the subject by announcing that he had discovered a special variety of bacillus, which he regarded as the true cause of the disease. Since that time, the interest taken in the subject by the medical profession has increased, not only on account of a more recent publication of Hansen, in which he claims the priority of discovery of the so called bacillus lepræ, but, furthermore, on account of the statements of two or three other observers, who also assert having seen the true bacillus of leprosy. During the year 1880, I had opportunities of studying the pathological changes, characteristic of leprosy, in three fatal cases of that disease, without, however, succeeding in discovering those granular masses enclosed within certain cells formerly described and regarded by Hansen as peculiar to leprosy; the later observations of this author, published in 1880, were then unknown to me. The results of my studies I embodied during the ensuing winter in a paper, published in the December number, 1881, of the "Archives of Medicine," and in which I also referred to the former observations of Hansen, while I hesitated to mention Neisser's bacillus for the want of confidence in the material in which he had discovered the organism. Thus matters stood, until not long ago, I read in some American medical journals that the bacillus lepræ had also, been seen upon this continent by Dr. I. Bermann, of Baltimore. This circumstance led me to make a careful re-investigation of the subject, based as in my former studies, upon a very close examination of a considerable number of very thin sections of leprous tissues and organs, treated with the famous bacteria detective, methylviolet. But as the principal object of this paper is to answer as satisfactorily as possible, the question by which it is headed, it becomes necessary to present to the reader some of the data relating to the history of the pretended parasites of leprosy, before stating the results of my more recent examinations, or before reviewing the statements of those observers who regard the pathological changes, characteristic of leprosy, as being caused by a special parasitic organism. For this purpose I shall principally make use of the numerous reports on leprosy, recorded in the "*Jahresbericht ueber d e Leistungen und Fastschritte in der gesämmten Medicin,*" edited by Profs. Virchow and Hirsch. To give to the reader an idea of the extent of the literature on leprosy, I may say that not less than 183 works and papers on this disease—including the Report of the Royal College of Physicians of London, 1867, which alone represents the facts extracted from 250 private reports—were reported in this great and unique journal since the year 1866. In nineteen of these reports or papers a microscopical examination of various tisues and organs had been made, though—excepting Hansen, Carter, Neisser, Hillairet and Gauché—none of the authors claim in any way to have met with bacteria in the tissues they examined. I may furthermore state that nearly every one of the authors of the above mentioned 183 reports—excepting again, Hansen, Carter and one or two others —pronounce the disease hereditary and not contagious.

Having satisfactorily introduced the subject by the above remarks, I shall briefly sketch the principal facts observed and relating to the parasitic organisms of leprous tissues, by simply quoting from the reports of the respective collaborators on leprosy in "Virchow and Hirsch's Jahresbericht," beginning with Hansen's first statements regarding the presence of bacteria in leprous tissues, reported in this journal, for the year 1870, vol. I. p. 257. In this report the following passage occurs: "When softening (emollition) of the tubercles takes place, peculiar elements appear, which the author, in his first article, regarded as being specific in nature, but which, in his second article, he attributed to the softening process. The latter may take a rapid course and appear one or two months before death, when all elements will be found disintegrating into fat and detritus; though it may also develop very slowly, in which case these peculiar elements will be met with as products of the retrogressive metamorphosis. With the beginning of the softening, the microscopical sections, show round, oval, spindle-shaped cells, with or without processes, which contain, besides the nucleus, one or more, larger or smaller, round, yellow, granular, small masses, not generally stained by carmine, though some of them may partially imbibe it. Sometimes they appear to be placed in a hollow space, frequently several of them fuse together within a cell, or are placed apparently free, as if escaped from the cells; their size ranges from that of a colorless blood corpuscle, to six or eight times as large; sometimes they fuse together into very irregular masses, exhibiting, frequently, semi-spherical prominences, which either appear to represent agglomerations of small masses, or to be surrounded by a colorless envelope. These masses, varying in their appearance and containing sometimes vacuole-like spaces, are represented by a number of drawings, etc."

In another paper, reported in the "Jahresbericht for the year 1874," vol. I, p. 441, Hansen states his reasons for regarding leprosy as contagious. The following passages occur in the reports: "The inoculation experiments, performed by the author have only given negative results. The author attaches no great importance to the examinations he made of the blood of lepers, but lays stress upon the constant occcurence of the 'brown elements' which he has formerly (1873) described, and which perhaps represents cells enclosing zoöglœa-masses. The culture experiments made with these small bodies—as well as with the rod-like bodies, also occurring in the tubercles—have given no definite results. To determine at present in what manner and by which elements, the disease is communicated, would be impossible."

The next allusion to the presence of parasitic organisms in leprous tissues appears to have been made by Carter in 1875, though there is no mention of his paper in the *Jahresbericht* for this year. The deficiency, however, is made up by a paper of G. Milroy, another known observer of leprosy, which we find reported (vol. I, p. 433) as follows:

"Milroy subjects to a critical examination the more recent views concerning the contagiousness of leprosy; he, especially, opposes the latest views of Hansen and of Vandyke Carter, who formerly was one of the most decided opponents of the doctrine of the communication of leprosy by contact, but who, of late, —led, as it appears, by the observations he made in Norway,—has made a turn to pass over into the camp of the contagionists. Milroy shows how little the facts, adduced by these observers in support of their views, will stand the test, and calls upon Carter, who has fallen a victim to the bacteria swindle, to furnish the proofs for the contagious importation of the disease to Honolulu, upon which he principally bases his view." To elucidate the preceding report I may remark that Carter is one of the most prominent contributors to the pathology and ætiology of leprosy; his observations were chiefly made in the East Indies, where, for many years, he was one of the strongest supporters of the hereditary and non-contagious nature of leprosy, but who, judging from the foregoing report, appears to have been induced by Hansen in Norway to embrace the doctrine of contagiousness and bacteria. It is thus that we find in the *Jahresbericht* for the year 1876, vol. I, page 373, his examination of the leprous spots mentioned as follows: "The histological examination of the diseased parts showed the generally unchanged skin and subcutaneous tissue densely infiltrated with a cellular mass, deposited alongside, and enveloping, not only the blood-vessels, but also the canals of the sweat glands, the hair follicles, the subcutaneous glands, and the nerves. Along and distributed between these elements were observed fine granular masses of a dark, orange-brown color, which, striated or aggregated, were, as it appeared, principally deposited along the lymphatics or smaller blood-vessels; upon the addition of acetic acid, a solution of carbonate of potassa, or sulphuric ether, they remained unchanged."

"In a second case of inveterate leprosy, in which the exanthema had appeared, the author had an opportunity to complete the above observations. Besides the before-mentioned pathological products, he found numerous accumulations of small spherical bodies arranged one along the other, the identity of which with micrococcus or zoöglœa masses became clear at the first glance. In dimension, form, and position these masses corresponded to lymphatic vessels, and a further examination showed, indeed, that they were deposited in these vessels, filling them almost completely; here and there the author met with larger empty spaces, corresponding to enlarged lymphatic vessels which had discharged their contents. There was nothing in the appearance of these embolic masses of micrococcus to distingish them from the ordinarily known forms; in the preparation examined they stood in intimate connection with the accumulations of the dark pigmented small bodies, so that, as the author suspects, this fresh leprous eruption has arisen from the remains of the former. The skin and its appendages were found perfectly normal."

The next observations of bacteria in leprous tissues are those of A. Neisser, the report of which, in the *Jahresbericht* for the year 1879, vol. I, p. 334, is as follows: "Neisser, in procuring preparations from leprous cadavers from Norway, found in all the fourteen pieces of skin and tubercles, in the liver, spleen, testicle, lymphatic glands and cornea, baccilli of the following description : Small, slender rods, measuring in length about half the diameter of a colored blood-corpuscle, their breadth corresponding to one-fourth of their length. They resemble nearest the small bacilli met by Koch (*Ætiologie der Wundkrankheiten*) in the septicaemia of mice, except that they were not so delicate as the latter. They could not be recognized in uncolored sections. Their arrangement corresponded to the place in which they had been developed. They rested either in numbers of two or three, one behind the other, representing an apparently long, sometimes curved thread, or six or seven of them placed alongside of one another in a regular parallel position ; or, they formed piles by overlying one another in all possible directions, the individual elements of which could only be resolved by a close exmination. In old preparations, that is, in leprous products of later stages, the rods appeared to have separated into granules. which, however, had retained their arrangement in rows. To regard the latter as micrococci the author rejects, but puts the question "whether the formation of granules did not rather signify a formation of spores as a retrogressive metamorphosis ?" In the skin the bacilli are equally distributed throughout all layers of the leprous infiltration ; in the testicle they formed a dense, easily crumbling mass, filling the seminiferous tubular-like casts, while in the lymphatic glands they were dwelling along the margin. In the ulnar nerves the rods were wanting. Of the two probabilities that the bacilli either existed primarily, or represented only a secondary accident upon a favorable nutritive soil, Neisser inclines to the former, because the presence of this unique variety of bacteria, together with its great distribution, spoke in favor of it ; though he is conscious that many clinical observations cannot be made to correspond with the view of leprosy being a bacteria disease."

In the *Jahresbericht*, etc., etc., for the year 1880, Vol. I, p. 388, we meet again with some communications on leprosy from Hansen, the report of which is as follows : "In the introduction to his explanation, regarding the 'Bacillus Lepræ,' Hansen first re-claims, in opposition to Eklund and Neisser, his priority. The statement of the former, that the minute rods, described at different times, could be readily found in the blood, he still—after perfecting his method of staining—rejects. In preparations of the blood of lepers which had been kept in the moist chamber, however, he found pointed filaments of a specific nature. In ten series of examinations into the microscopical nature of the tubercles, Hansen always could show with greater facility those conglomerates he formerly described—masses of zoöglœa or collections of bacilli enclosed in cells—than to obtain an entirely satisfactory view of the arrangement and form of those rods. They were, however, present in the great majority of the preparations, while the presence of the jointed filaments was very irregular. Only, finally, with the assistance of the more intense (methylviolet) staining, has he succeeded in showing the rods, also, in sections of the tubercles hardened in absolute alcohol. As regards their signification of representing the true infections, or contagious matter of leprosy, Hansen expresses himself with a praiseworthy caution. This time, as before, he never obtained positive results, regarding contagion, by repeated subcutaneous incorporation of the matter of tubercles into rabbits."

In the same volume of the *Jahresbericht* we also find reported an observation, made by Hillairet and Gauché, concerning the presence of bacteria in the blood of a woman who had lived in the Cordilleras, before she came to Paris. I omit to cite this observation on account of its apparent unreliability.

In the *Medical News* of January 7, 1882, a communication from a correspondent in Baltimore to this journal will be found, which, for the purpose of referring to some special data it contains, I shall quote in full, as follows: "The Bacillus Lepræ.—The recent descriptions of Neisser, Eklund, and others, of the bacillus lepræ, first observed by Hansen, have awakened widespread interest in the pathology of leprosy. Dr. I. Bermann, of this city, has been able to detect the presence of the bacillus in leprous tissue, and to confirm the discoveries of the above-mentioned authors. Sections exhibiting the bacillus were shown at a recent meeting of the American Dermatological Association at Newport. Since then Dr. Bermann has improved his methods of preparation and investigation, and is now able to demonstrate the bacillus to perfection. His results were shown at a recent meeting of the Baltimore Clinical Society. The directions given by Neisser for detecting the little organisms enable one to recognize them, but not with satisfaction. Weigert's method for investigating bacteria answer the purpose perfectly. The affinity of the bacillus lepræ for the aniline violet staining fluid is remarkable. By immersing the stained sections in oil of cloves for 48 hours, the violet color is removed almost completely from the tissues, leaving the bacilli stained dark purple and presenting sharp outlines. It remains very difficult to see them, however, even after this preparation, and objectives of high power (with small angle) must be employed. By using as a condenser, an objective of about one-quarter inch, for illumination, they come out with splendid definition. The bacilli are mostly to be found in the protoplasm of the cells, but may be seen elsewhere. Sections thus prepared and examined will satisfy the most incredulous. Dr. Bermann has been the first in this country, we believe, to detect the bacillus, and will shortly publish his observations in full."

Postponing for the present the review of the above-cited observations of Hansen, Carter, Neisser and Bermann, I shall first proceed to state the results of my recent examinations of a considerable number of sections of different leprous organs. These sections were cut during the summer, and following fall and winter of 1880, and since that time kept in a mixture of alcohol and filtered rain-water—50 per cent. of each—affording the sections, as may be readily understood, every opportunity of being invaded by bacteria, bacilli, or other fungi. A number of them, representing the skin, epiglottis, liver, kidney, lymphatic glands, supra-renal badies, etc., etc., were put into a strong alcoholic solution of methylviolet, half dilùted with water, and left there for about sixteen hours, so that, when removed from this solution, they were found intensely stained; others, put into a watery solution of methylviolet, were found equally stained. After their removal from the staining fluid, the sections were put, first in commercial, and then in absolute alcohol, which caused them to part with the methylviolet they had absorbed almost entirely. Nevertheless, in order to follow closely Dr. Bermann's directions, they were then put in the oil of cloves—for which, however, very little color had remained to be extracted—and left there (unnecessarily) for from 24 to 48 hours, to be finally mounted in Canada balsam. In subjecting these sections to a close microscopical examination, I failed, notwithstanding their remarkable thinness and transparency, to detect even a single bacillus. The only parasitic organisms met with were an inconsiderable number of patches, or so-called zoöglœa of micrococcus, thoroughly stained by the bright blue of the methylviolet; but these, even, were principally confined to the sections of skin taken from the lobules of the ear from the first and third cases. The most of these patches were small, and ellipsoidal, or spindle-shaped in form, the smallest corresponding in size and outline to the larger or smaller spindle-shaped cells met in the corium of the skin, while the larger ones were generally composed of a number of the former, either placed one behind the other, or, in some instances, overlapping one another. In most instances, the long axis of these ellipsoidal, or spindle-shaped zoöglœa, was placed parallel with the surface of the skin, or with the course of the connective tissue bundles of the corium, though others were observed placed obliquely, or, also, to present irregular outlines. A few larger patches, round or oval in outline, and corresponding to the shape of a transverse, or oblique section of a larger vessel, were likewise met with. In all these patches of micrococcus, however, the individual micrococci could be distinctly recognized, particularly by their bright blue color, contrasting with the very feeble picro-carmine staining of the sections; the original structure of the latter showed as perfectly as 15 months before, when the sections were cut. In the skin, taken from the second case, no trace of microccus, even, could be detected.

But, as regards the micrococus-zoöglœa observed upon the sections of skin, it must be remarked that they were only found upon the corium of the skin on one of the lobule, the other side being perfectly free, and, by alternately changing the focus, it was furthermore found that the patches of micrococci rested almost always upon one, or the other surface of the section; in a few ex-exceptional instances, only, the minute patch appeared to pass into the section. But, even in these instances, a closer examination revealed them deposited in some interspace of the connective tissue-bundles, or, in a vessel laid open by being cut in an oblique or longitudinal direction. The ellipsoidal, or spindle-shaped form, which, perhaps, the greater part of these patches or zoöglœa, presented, was very probably caused by the settling of the first individual micrococci upon one of those numerous spindle-shaped cells, met with in the corium, or upon a connective tissue bundle very obliquely cut, in consequence of which the patch, arising from those first settlers, finally assumed the form of the host upon which it grew. In the same manner, the colony may, in other instances, have assumed the form of a vessel by having settled and grown into the lumen laid open by the knife. Those zoöglœa of a round or oval form, generally larger in size, and by their greater thickness less transparent, were most probably deposited in the transversely, or obliquely cut lumen of a large vessel. Now, whether the ellipsoidal, or spindle-shaped forms of the patches were really determined by the causes I have pointed out above, or whether the latter assumed these forms independently of these circumstances, I would not positively assert, though the explanation I have offered appears to be the most plausible. Neither do I deem it necessary to further speculate, in this place, as to the cause of the irregular forms, or those patches placed obliquely to the course of the fibrous bundles of corium; though, before dismissing the skin, I repeat: *that in no instance did I ever observe micrococci lodged in the interior of any of the cells*, notwithstanding the sections being sufficiently thin to have shown the parasites, even without staining.

The next, and only remaining instance, in which I met with micrococci, was in a small number of empty uriniferous tubules, contained in some exceedingly thin sections of the kidney taken from the first case. In these tubules, represented only by the basement membrane, the layer of micrococci, if such they were, was very thin, and presented a very faintly-colored appearance, so that the question may still be asked, whether these faint, granular masses might not represent degenerating, granular, urinary cylinders. At any rate, the tubules had been laid open by the knife, affording an opportunity to the parasites to found a colony in the lumen of the former. In these sections, also, not a trace of a bacillus form could be discovered.

Finally, the exceedingly fine filaments of a fungus, about $\frac{1}{1600}$ m.m. in thickness remain to be mentioned ; they were observed to spread *over* some sections of tongue, liver, kidney, and lymphatic gland, but in no instance entered the tissue ; on the contrary, in order to render these filaments distinct, the objective had to be adjusted so far away from the section as to bring the histological elements of the latter entirely out of focus.

It appears to me almost superfluous to enlarge to any extent upon the signification of the presence of the micrococcus-zoöglœa, or upon that of the fine fungous filaments, found, as stated above, upon some of the sections, for it is obvious that they are deposited after the latter were cut. And, in regard to the fungous filaments, I hestitate not to to say that, being very readily stained by hæmatoxylin, I have observed them well enough during my first examinations in the pathology of the disease—but, knowing, at that time, that they bore no relation whatever to the tissues I was then studying, it never entered my mind to create a sensation by mentioning them in my paper. At what particular period of the process of preparing the sections they found their way upon the latter, I am unable to say, but it must be known to every practical histologist that solutions of carmine, or of hæmatoxylin, do frequently attract fungi, and that, in the above case, the germs may have settled upon the sections while lying in the staining fluid, or, subsequently, in the mixture of alcohol and water, in which I generally preserve my sections before they are wanted. The micrococci, found upon the sections which I recently examined, may have been deposited since the time of my first examination—that is, while they were kept—each kind of section in a separate bottle—in the alcoholic solution. This becomes the more probable in calling to mind the sections of skin, upon which the micrococci colonies were only found deposited upon one-half of the surface, for the reason that the other half was very likely protected by another section lying upon it.

In summing up, now, the above stated results of my recent examinations of leprous tissues, we find that in these three fatal cases of leprosy, which came under my observation, the "Bacillus lepræ" could not be found, a circumstance which justly entitles us to entertain serious doubts as to its real existence. But before further commenting upon the subject, let us review the statements of those observers who claim to have beheld this parasitic organism. Thus, in referring to the statements of Hansen, made 1870, and cited in the beginning of this article, it will be noticed that the "peculiar elements" appearing in the course of the softening of the tubercles—and which this author formerly regarded as specific in their nature—he now attributes to this process, and regards them as products of a retrogressive metamorphosis. The granular masses to which he refers, and which he describes as not generally stained by carmine, though some of them may partially absorb it, I am inclined to identify with those large groups of more or less degenerated neoplastic cells, originally derived from the nuclei of the fat-cells, and replacing the latter. These groups are mostly found lying free in the areolar spaces of the corium, or subcutaneous connective tissue, and, in accordance with the degree of degeneration which they have undergone, either absorb or refuse the carmine. In my paper on the pathologicel anatomy of leprosy, I have sufficiently described the groups of cells, but have, unhappily, omitted to mention certain collections of very fine, slightly curved and sword-shaped crystals of margaric acid which I observed in many of these degenerating, compound cellular bodies. These crystals, though easily recognized, might nevertheless, in sections not stained with hæmatoxylin, or methylviolet, be taken for bacilli. In many of these cellular masses some of the nuclei are still observed, especially if the preparation has, after the picro-carmine staining, been feebly stained with hæmatoxylin, such as I have indicated in my paper. In other instances the nuclei have entirely disappeared by the advanced degeneration of the cells, and nothing but the fine irregular outlines of the latter are left, the whole mass being stained yellow by the picric acid.

There are no special remarks to be made upon the statements of Carter, as they clearly show that the minute organisms which he observed presented—as he states himself—nothing to distinguish them from the ordinarily known forms of micrococcus. Though I know nothing about the manner in which Carter preserved and prepared his leprous tissues, I nevertheless presume that his micrococcus lepræ—if such we may call it—is identical with the one which I met in my recent examinations, and may be traced back to the same origin. The former observations of Hansen with those of Carter, therefore, do not present the slightest fact to justify the theory of the parasitic origin of leprosy.

In reviewing the observations of Neisser, it appears from his description that he had real bacilli under his microscope. The presence of these organisms in the leprous tissues which he examined, however, loses very considerably in significance by a consideration of the manner in which he procured his material, as we do not know how many hours after death these tissues were removed from the cadaver, and how many chances they had of being invaded by bacilli, before they even reached the hands of the investigator. But in comparing the statements of Neisser with the more recent observations of Hansen, the probability of the presence of bacilli in the tissues they examined gains somewhat in strength, though I am still far from understanding how these organisms made their way into the interior of the cells. The statement of Neisser that in leprous products of later stages the bacilli should have resolved into granules—which, having retained their arrangement in rows, he regards as spores—throws much doubt upon the exactness of his examinations, for it is clear that these granules represented sphero-bacteria, such as they are often observed in the vicinity of micrococcus-zoöglœa. The same may be said of the dense, crumbling masses, filling up the seminiferous tubules like casts. On the whole, Neisser's view, that the presence of these organisms in the leprous tissues is primary, certainly rests on a very weak foundation, rendered more apparent by his acknowledgment of being conscius that the clinical history of leprosy cannot be brought into harmony with the prevailing bacteria theories. Hansen's observations, on the other hand, appear to be more trustworthy, as he freely acknowledges the existing defects of the observations, stating, for instance, that he could not obtain an entirely satisfactory view of the arrangement and form of the rods ; the presence of the rods in the blood of lepers he positively denies. The observations made on blood kept in the moist chamber I regard as unreliable, for the reason that the minute organisms are found everywhere, very frequently in places where no account can be rendered for their presence. The various attempts made by Hansen for the purpose of producing the disease in rabbits by inoculation of leprous matter, have, as we have seen, also failed.

Finally, in the report of Dr. Bermann's observations, regarding the existence of the bacillus lepræ, we meet with the same flaws as in those of the preceding observers. The very confession, made in this report, that even with the methylviolet staining, it still remained very difficult to see the bacilli, and that an objective of high power with small angle must be employed, renders the whole observation doubtful; for I may safely assert that if the bacilli are really present in the section, and the latter is sufficiently thin, they can be distinctly seen—particularly when stained with the mithylviolet—even with an amplification as low as from 300 to 350 diameters. Neither is it necessary to employ a condenser, for a good mirror with a clear, bright daylight, and a small diaphragmatic opening closely applied to the object, will answer all purposes. The only essential condition is that the sections be sufficiently thin to be rendered perfectly transparent. Anyone interested in the subject may convince himself of these facts by putting a small quantity of hay into a wide-mouthed bottle containing water, and setting it aside in a warm place. After the lapse of a few days a delicate pellicle will appear upon the surface of the solution, and, if a drop of the latter with a minute portion of the former is examined under the microscope, bacteria and bacilli of various kinds will be met with, and distinguished without difficulty, with the powers I have mentioned. To render them, however, still more distinct, a drop of the solution may be spread upon a glass slide and left uncovered until nearly evaported, when a drop of a solution of methylviolet is put upon the residue, and a thin cover-glass made to rest lightly upon it. Before long the organisms will have absorbed sufficient staining material as to appear still more distinct under the microscope. Thus, it will be found that the detection of these minute organisms is by no means as difficult as some enthusiastic germ-theorists represent it. In fact, there are many objects met with, both in normal and pathological histology, the nature and relationship of which are much more difficult to determine than to recognize a bacterium or bacillus.

But let us return once more to the bacilli met with in the leprous tissues by the above-mentioned observers, and, while admitting the truth of their statements, ask for the signification of the phenomenon, or its bearing upon the origin of leprosy. There is no other answer to this question, but a simple denial of any relationship existing between these organisms and the original cause of leprosy, until they can be shown in the blood of *every case* of this disease, and, more, *in such numbers* as to render their noxious influence upon the human organism evident. We may furthermore ask an explanation for the total absence of bacilli in the numerous sections—taken from the organs of three typical cases of leprosy—which I examined, even with the assistance of the bacteria-detective, the methylviolet. The discrepancy existing between the results I obtained myself and the statements of the above observers, can only be explained by either presuming that some mistake has been made on one side or the other, or by supposing the bacilli to have entered the tissues from without. The latter may take place through an ulcerated tubercle, or by the deposition of some of the organisms upon the sections in the manner I have explained above; or, also, the bacilli may have found access into the pieces of tissue after these were removed from the cadaver and before the cutting of the sections; this supposition is applicable to the tissues Neisser had before him. There are a number of ways by which bacilli may enter the tissues, leaving aside the possibility of these organisms arising as products of the disease in the living tissues themselves, a view which does not lack supporters. At any rate, the tissues which I examined were tolerably fresh, the autopses having been made but a few hours after death.

In conclusion, I may say that the opposition, taken by me in the above review against the theory that leprosy is caused by minute parasitic organisms, is not based upon pre-conceived notions; on the contrary, I am far from denying the possibility of bacilli causing certain diseases, as, for instance, splenic fever, though here, it must be remembered, the bacilli are found in the blood itself, or to obstruct the smaller blood-vessels and lymphatics in the form of emboli, while, as far as I know, they have never been observed in the protoplasm of cells. But admitting, even, that such a phenomenon actually presented itself in leprosy, its explanation, that is, the way by which these organisms entered the interior of the cells, would still remain obscure unless we take recourse to the supposition that they originated in the protoplasm itself. Excluding, however, their origin within the tissues, there remain only two ways by which they could enter the human organism ; these are, either through an open lesion, or by entering the blood through the lungs or alimentary canal ; and, it is not at all improbable that in some instances they may enter through an open lesion, especially at the time when the tubercles commence to undergo softening and ulceration, such as Hansen has stated. Their presence in the blood this author has repeatedly denied. In my own examinations I have never observed the slightest trace of a bacillus in the smaller blood-vessels, notwithstanding the sections examined were sufficiently thin for recognizing with the greatest distinctness the blood-corpuscles contained within. Besides, the clinical history of leprosy is so well known that I may refrain from further showing the difficulty of making the characteristic symptoms of this disease harmonize with the bacteria theory ; in every instance where bacteria or bacilli are really met with in the tissue it must be presumed that they arrived there by accident and in search of a nutritive soil for their development. But as it is desirable that the truth should be ascertained, and as I fortunately still have numerous sections of various leprous tissues—kept in alcohol and water—on hand, I shall be pleased to furnish, upon application, a number of them to any competent pathologist, who would like to investigate the subject with the view of publishing the results of his examinations.

---

A number of microscopical preparations taken from each of these cases, accompanicd this paper.

Dr. Schmidt requested that a committee be appointed to examine them in order to determine whether they would substantiate the statements which he had made concerning them. The following committee was therefore chosen, who reported as follows at the meeting of the society :

"The committee appointed for the purpose would report that they have examined the slides accompanying Dr. Schmidt's paper, and would say that they have been unable to find any bacilli in them. The slides appear to agree in every respect with Dr. Schmidt's statements in regard to them.

"LESTER CURTIS, M.D.,
"Prof. of Histology, Chicago Medical College.
"JAMES NEVINS HYDE, M.D.,
"Prof. Dermatology, Rush Medical College.
"CHRISTIAN FENGER, M.D.,
"Pathologist to Cook County Hospital."

---

